# Combining standard clinical blood values for improving survival prediction in patients with unresectable esophageal carcinoma: A retrospective study

**DOI:** 10.1097/MD.0000000000044451

**Published:** 2025-09-12

**Authors:** Lei Zhao, Dan Mei, Xiaolin Ge, Yujing Shi, Xinchen Sun, Liang Liang

**Affiliations:** a Department of Oncology, Jurong Hospital Affiliated to Jiangsu University, Zhen Jiang, China; b Department of Clinical Research Coordination, Nanjing Yuzhong Medical Consulting Co., Ltd., Nanjing, China; c Department of Radiation Oncology, The First Affiliated Hospital of Nanjing Medical University, Nanjing, China.

**Keywords:** concurrent chemoradiotherapy, esophageal squamous cell carcinoma, LabBM Score, nutrition and inflammatory markers

## Abstract

The prognosis for patients with esophageal squamous cell carcinoma (ESCC) is poor, with a 5-year survival rate of approximately 30%. There are significant differences in prognosis among patients, and some patients experience local recurrence and distant metastasis within 1 year after comprehensive treatment. Therefore, there is an urgent need for highly sensitive and specific biomarkers to assess the therapeutic risks and prognosis of patients with inoperable esophageal cancer. This study aimed to investigate the predictive value of the LabBM score in the survival of patients with inoperable ESCC. A single-center retrospective study was conducted, including 150 and 155 patients with unresectable ESCC who received concurrent chemoradiotherapy at our hospital between January 1, 2013, and June 30, 2018, and between July 1, 2018, and March 1, 2020, respectively, as the training and validation cohorts. Laboratory parameters, including lactate dehydrogenase, platelet count, C-reactive protein, and albumin, were collected 1 week prior to concurrent chemoradiotherapy. The LabBM score was calculated, and survival differences were analyzed across patients with different LabBM scores. Cox regression and Kaplan–Meier analyses were used to assess factors influencing progression-free survival (PFS) and overall survival (OS), aiming to clarify the prognostic value of the LabBM score for PFS and OS. In the training group, of the 150 patients enrolled, 123 were in the low LabBM group (0–1), 23 in the medium LabBM group (1.5–2), and 4 in the high LabBM group (2.5–3.5). The median survival was 33 months in the low LabBM group, 11 months in the medium LabBM group, and 6.5 months in the high LabBM group, with a significant difference in survival between the 3 groups (*P* = .000). Univariate Cox analysis showed that a higher LabBM score was associated with worse OS (*P* < .05). Multivariate Cox regression analysis revealed that a higher LabBM score (hazard ratios: 2.75, 95% CI: 1.08–7.03, *P* = .034) was an independent influencing factor for OS. In receiver operating characteristic analysis, the AUC area for OS and PFS predicted by the LabBM-based risk model was 0.92 (95% CI: 0.86–0.97, *P* = .00) and 0.956 (95% CI: 0.88–0.98, *P* = .000), respectively. The AUC for LabBM scores predicting OS and PFS were 0.63 (95% CI: 0.54–0.72, *P* = .011) and 0.61 (95% CI: 0.52–0.70, *P* = .039), respectively. The AUC area of the risk model was significantly higher than that of other single parameters. Subsequently, we re-enrolled 155 patients with inoperable esophageal cancer in different time periods for the above analysis, and the results were consistent with the experimental group. In inoperable ESCC patients, the LabBM score-based risk model is a novel and effective prognostic indicator.

## 1. Introduction

Esophageal cancer is the 6th most lethal tumor in the world^[1]^ and has a high incidence in Asia, especially in China. Esophageal cancer is mainly classified as squamous and adenocarcinoma. More than 90% of patients in China with esophageal cancer are esophageal squamous cell carcinoma (ESCC).^[2,3]^ Since patients with early ESCC do not have typical symptoms, most ESCC patients are detected at an advanced stage and often have poor prognoses.^[4,5]^ Surgery, radiotherapy, and chemotherapy are the main treatments for ESCC. However, for patients with locally advanced ESCC, concurrent chemoradiotherapy (CCRT) remains the first choice, with the advantage of common side effects and high overall patient survival.^[6]^ In recent years, targeted therapy and immune checkpoint inhibitors have been widely used by physicians, and conformal radiation techniques and radiation dose rates have also been greatly improved, which has led to a significant improvement in the efficacy of CCRT with manageable side effects.^[7,8]^ However, the prognosis of ESCC patients has not improved significantly, with a 5-year survival rate of only about 30%. In addition, there were significant differences in outcomes between patients. There are even some patients who develop local recurrence within 1 year.^[9–11]^ Currently, the TNM staging system is considered the best clinical survival prediction standard and is widely used in clinical practice. However, it has low sensitivity and specificity for early diagnosis or recurrence.^[8]^ Therefore, there is an urgent need for highly sensitive and specific biomarkers in clinical practice for risk assessment of treatment and prognosis of patients with inoperable esophageal cancer.

Several studies have demonstrated that the oncology patients’ nutritional and inflammatory status is significantly associated with their prognosis. The biomarkers in blood samples can reflect the nutritional and inflammatory status. Also, as a noninvasive test, blood sample testing has the advantages of easy detection, low cost, high patient acceptance, and facilitates the early detection and follow-up management of cancer.^[12–17]^ A study constructed a unique prognostic model using indicators from blood sample testing (hemoglobin, platelets, albumin, C-reactive protein [CRP], and lactate dehydrogenase [LDH]) called the LabBM score. This study demonstrated that the LabBM score could predict the survival of patients with brain metastases.^[18,19]^ In a subsequent study, Niederer C et al also showed that the LabBM score was a good predictor of prognosis in patients with stage II to III non-small cell lung cancer.^[20]^ The higher the LabBM score, the worse the prognosis of the patients. There is no report of LabBM score predicting the prognosis of patients with esophageal cancer. Therefore, we collected clinical data with inoperable ESCC who received CCRT at our center to clarify the prognostic, predictive value of the LabBM score in this group of patients.

## 2. Materials and methods

### 2.1. Study subjects

Patients with ESCC who received CCRT at our hospital were enrolled in this study. Patients treated between January 1, 2013 and June 30, 2018, were assigned to the training cohort, while those treated between July 1, 2018 and March 1, 2020 were assigned to the validation cohort. We declared that all relevant procedures were approved by the Institutional Review Board and performed in compliance with the Helsinki Declaration. This research was supported by the Medical Research Ethics Committee of Jiangsu Provincial People’s Hospital Jurong Branch (No. JRSRMYY-2023-063), all subjects signed informed consent and agreed to take part in this study. Inclusion criteria: inoperable locally advanced ESCC (T2-4N0-+M0-1) as assessed clinically and imaging, or with contraindications to surgery; receiving targeted CCRT and not undergoing surgery; hematological examination within 1 week before the first radiotherapy; Karnofsky Performance Status (KPS score 60–100); age 18 to 80 years. Exclusion criteria: combined other malignant tumors; contraindications related to radiotherapy; patients with brain metastases; severe infectious diseases and hepatitis cirrhosis; incomplete clinical and follow-up information.

### 2.2. Treatment regimen

All patients were irradiated with 6⁃MV X-ray linear acceleration at a dose of 60 to 66 Gy (2.0 Gy/fraction, 5 times/week); 2 cycles of synchronous chemotherapy were administered with the following regimens: RP regimen (raltitrexed 2.5 mg/m^2^ d1 + cisplatin 25 mg/m^2^, d1–3) and TP regimen (doxorubicin 60 mg/m^2^ + cisplatin 25 mg/m^2^, d1–3). All patients were evaluated with barium fluoroscopy, serum tumor markers and cervical, chest, and abdominal enhancement CT scans 1 month after synchronous radiotherapy. The results were recorded for the study.

### 2.3. LabBM score definition

Included patients were required to undergo the blood routine examination, comprehensive metabolic panel, and CRP 1 week before CCRT to calculate LabBM score (institutional normal values: hemoglobin [Hb] 11.7–15.3 g/dL (women) and 13.4–17.0 g/dL (men); platelets (PLT) 130-400 × 10^9^/L; albumin 34–45 g/L; LDH < 255 U/L; CRP < 5 mg/L). The scoring criteria, referring to previous studies, were 1 point for LDH and CRP above the upper limit of normal, and 0.5 points for hemoglobin, platelets, and albumin below the lower limit of normal, respectively.^[19]^ In this study, patients were stratified based on their LabBM score into the following groups: low LabBM score group (L-LabBM group) (0–1), middle-LabBM score group (1.5–2), and high LabBM score (H-LabBM) group (2.5–3.5).(Table 1).

**Table 1:** The components of the LabBM score.

	Institutional normal values	Abnormal	Score
C-reactive protein (CRP)	<5 mg/L	≥5 mg/L	1
Hemoglobin (Hb)	11.7–15.3 g/dL (women) 13.4–17.0 g/dL (men)	<11.7 g/dL (women) <13.4 g/dL (men)	0.5
Platelets (PLT)	130–400 × 10^9^/L	<130 × 10^9^/L	0.5
Albumin (ALB)	34–45 g/L	<34 g/L	0.5
Lactate dehydrogenase (LDH)	<255 U/L	≥255 U/L	1

### 2.4. Follow-up visits

Follow-up was conducted through outpatient visits or telephone interviews. During the first 2 years after completion of chemoradiotherapy, follow-ups were performed every 3 months, and then every 6 months thereafter. The follow-up period concluded in June 1, 2021 and Septemper 30, 2022, respectively. Follow-up assessments included laboratory tests (complete blood count, biochemical tests, CRP, and serum tumor markers), upper gastrointestinal imaging, and contrast-enhanced CT scans of the neck, chest, and abdomen. The median follow-up durations were 27.5 and 22.8 months (range: 1.0–81.0 months), with no patients lost to follow-up. Overall survival (OS) was defined as the time from CCRT to death from any cause, while progression-free survival (PFS) was defined as the time from CCRT to either tumor progression or death from any cause.

### 2.5. Statistical methods

SPSS 23.0 (IBM SPSS Statistics, Armonk: IBM Corp.) and GraphPad (GraphPad Software, Boston) software were used to complete the statistical analysis. Categorical data were compared between different subgroups using Pearson chi-square test or Fisher exact test. Survival analysis for OS and PFS was performed using the Kaplan–Meier method, and differences in survival were assessed using the Log-rank test. Cox proportional risk models were used to detect risk factors associated with OS and PFS, and the results of hazard ratios (HR) with 95% CI for these models were described. Subject work curve analysis (receiver operating characteristic [ROC]) was performed to assess the prognostic assessment ability of each parameter, including LabBM score, The area under the ROC curve (AUC) for each parameter was compared, and the Z-test was used to assess differences between the AUCs of various indicators. Stata software was used to build and validate models identifying independent prognostic factors. Clinical decision curve analysis and ROC curves were employed to evaluate the clinical utility of the risk model. A 2-sided *P*-value of < .05 was considered statistically significant. The datasets generated and/or analyzed during the current study are available in the https://www.jianguoyun.com/p/DYfWBa0QgcLUChj5-rYFIAA.

## 3. Results

### 3.1. The training group characteristics

One hundred fifty patients were included in this study for analysis, 125 (83.3%) of which were male and 25 (16.7%) were female. The median age at diagnosis was 65 years (37–79 years). The percentage of primary sites of lesions: upper esophagus (cervical + upper thoracic) accounted for 48.0% (72/150), middle esophagus (mid-thoracic) accounted for 28.0% (42/150), and lower esophagus (sub thoracic + abdominal) accounted for 24.0% (36/150). The median length of the primary tumor was 6 cm. According to the 8th edition of AJCC clinical TNM staging,^[21]^ the percentage of patients with stage II, III, and IVA was 16.7% (25/150), 2.7% (4/150), and 80.6% (121/150), respectively. Among the enrolled patients, 25 received only 1 cycle of concurrent chemotherapy because they could not tolerate the side effects of CCRT. Sixty-seven patients received 2 to 4 cycles of consolidation chemotherapy with the same regimen after completing CCRT. The number of patients with degrees I to II and III to IV myelosuppression during CCRT was 93 and 50, respectively. Thirty patients had KPS scores ≤ 80 and 120 patients had KPS scores > 80 (Table 2).

**Table 2:** Training cohorts patients’ information.

Characteristics			
Gender		Lymph node metastasis (N-stage)	
Male	125	N0	85
Female	25	N1	35
		N2	17
		N3	13
Age (yr)		cTNM stage	
Range	37–79	ⅠI	18
Median	65	III	11
		IVa	121
KPS score		Metastasis	
<80	30	Yes	64
≥80	120	No	86
Location		Length	
Upper	72	≥5 cm	115
Middle	42	<5 cm	35
Lower	36		
Tumor invasion (T stage)		Myelosuppression	
T2	19	I–II°	97
T3	14	III–IV°	53
T4	117		

### 3.2. The training group relationship between LabBM scores and clinicopathological characteristics

By analyzing the LabBM score and clinicopathological factors, we found that PLT as a constituent parameter of the LabBM score was not significantly correlated with LabBM. In contrast, the rest of the parameters (LDH, CRP, Hb) and gender were significantly associated with LabBM composition (*P* < .05). There was no significant correlation (*P* > .05) with age, T stage, N stage, clinical stage, KPS score, tumor length, and tumor location (Table 3).

**Table 3:** Training group baseline characteristics, treatment, and laboratory data according to LabBM score (N = 150).

Characteristics	L-LabBM (123)	M-LabBM (23)	H-LabBM (4)	*P*
Gender				**.051** **†**
Male	99	22	26	
Female	24	1	0	
Age (yr)				.50†
≥65	64	12	1	
<65	59	11	3	
KPS				.92†
<80	28	7	4	
>80	95	16	0	
T stage				.14†
T2	18	1	0	
T3	12	1	1	
T4a	93	21	3	
N stage				.87†
N0	71	10	4	
N+	52	13	0	
cTNM stage				.46†
II	22	2	1	
III	4	0	0	
IVA	97	21	3	
Length				.75†
≥5 cm	95	17	3	
<5 cm	28	6	1	
Location				.90*
Upper	57	14	1	
Middle	36	5	1	
Down	30	4	2	
PLT				.05†
NR	120	22	3	
>ULN	3	1	1	
ALB				
NR	116	18	2	**.00** **†**
>ULN	7	5	2	
Hb				
NR	74	2	0	**.00** **†**
>ULN	49	21	4	
LDH				
NR	115	15	1	**.000** **†**
>ULN	8	8	3	
CRP				
NR	118	10	0	**.000** **†**
>ULN	5	13	4	

Bold values indicate that the difference is significant, with a *P*-value <.05.

ALB = albumin, CRP = C-reactive protein, Hb = hemoglobin, H-LabBM = high LabBM score, KPS score = Karnofsky Performance Status, LDH = lactate dehydrogenase, L-LabBM = low LabBM score, M-LabBM = middle LabBM score, PLT = platelets.

* Chi-square test.

† Fisher exact test.

### 3.3. The training group effect of LabBM score on prognosis

#### 3.3.1. OS

**Figure 1.**
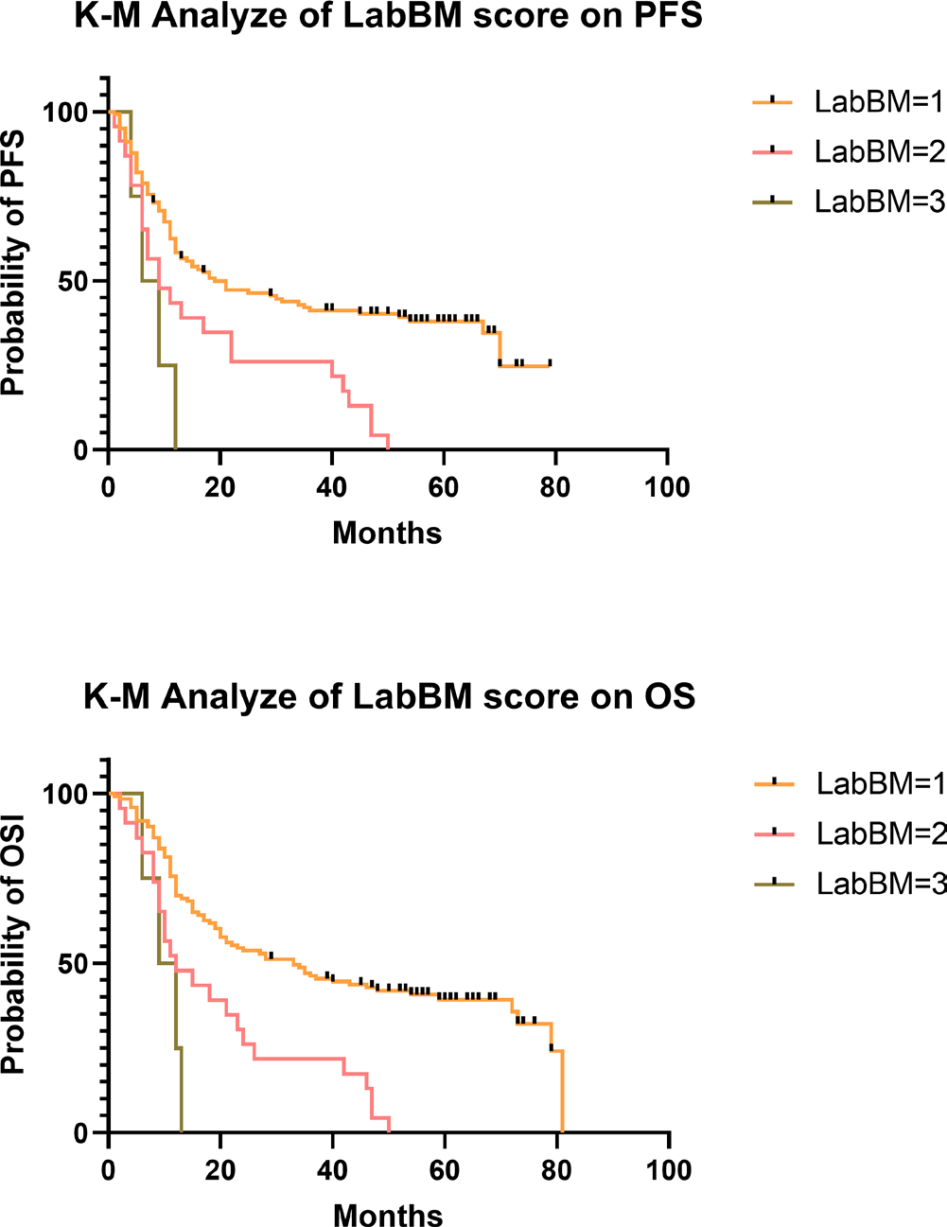
Differences in survival of different groups of LabBM score in the training cohorts (n = 150) (OS *P* = .000, PFS *P* = .00). OS = overall survival, PFS = progression-free survival.

Further analysis was performed according to the distribution of patients’ LabBM scores. The results revealed differences in OS among the 3 groups of patients with scores of 0 to 1, 1.5 to 2, and 2.5 to 3.5. The final results showed 123 patients in the L-LabBM group (0–1), 23 in the medium LabBM (M-LabBM) score (1.5–2), and 4 in the H-LabBM score (2.5–3.5). Median survival was 33 months in the L-LabBM group, 11 months in the M-LabBM group, and 6.5 months in the H-LabBM group, with a significant difference in survival between the 3 groups (*P* = .000) (Fig. 1 lower). Thus, our results demonstrate that the higher the LabBM score, the worse the OS of patients (*P* < .05). We found that patients’ OS was negatively correlated with LabBM, consistent with previous studies.^[18,20]^

We performed univariate and multivariate COX regression analyses to clarify the relationship between LabBM and OS. Univariate COX analysis showed lower KPS score, higher LabBM score, pretreatment metastasis, failure to achieve CR or PR after treatment, higher T stage, N+, higher TNM stage, lower serum albumin level, and pretreatment anemia were associated with poorer OS (*P* < .05). At the same time, there was no significant correlation with tumor length, tumor location, myelosuppression, grade of myelosuppression, age, gender, total radiotherapy dose, number of simultaneous chemotherapy, number of consolidation chemotherapy, and chemotherapy regimen were not significantly correlated (*P* > .05). Multivariate COX regression analysis showed higher LabBM score (HR 2.75 [1.08–7.03] *P* = .034), pretreatment metastasis (HR 3.45 [2.11–5.63] *P* = .000), failure to achieve CR or PR after treatment (HR 2.93 [1.59–5.36] *P* = .001), and lower KPS score (HR 1.64 [1.15–2.34] *P* = .006) were independent high-risk factors for patients’ OS (Table 4).

**Table 4:** Univariate and multivariate analysis of overall survival based on clinicopathological characteristics (N = 150).

Characteristics	Univariable		Multivariable	
	HR with 95% CI	*P*-value	HR with 95% CI	*P*-value
Age (yr)	1.35(0.92–1.99)	.13		
≤65				
>65				
Gender	0.64 (0.36–1.1)	.12		
Male				
Female				
Location	1.06 (0.83–1.34)	.65		
Upper				
Middle				
Down				
KPS	1.72 (1.2–2.4)	**.001**	1.64(1.15–2.3)	**.006**
<80				
≥80				
Chemotherapy regimen	1.13 (0.77–1.7)	.53		
TP				
RP				
Lymph node metastasis (N-stage)	1.59 (1.08–2.4)	**.019**	1.35 (0.88–2.08)	.17
N0				
N+				
Tumor invasion (T stage)	1.76 (1.2–2.5)	**.002**	1.4 (0.7–2.7)	.37
T2				
T3				
T4				
TNM stage	1.5 (1.1–2.06)	**.01**	0.89 (0.49–1.5)	.68
II				
III				
IVa				
Length	0.74 (0.46–1.2)	.21		
<5 cm				
≥5 cm				
Dose	1.39 (0.57–3.5)	.47		
<60 Gy				
≥60 Gy				
ALB	2.4 (1.35–4.2)	**.003**	1.25 (0.64–2.4)	.51
NR				
ULR				
LDH	1.89 (1.1–3.2)	.015		
NR				
ULR				
PLT	0.73 (0.23–2.31)	.59		
NR				
ULR				
Hb	1.51 (1.02–2.2)	**.039**	1.06 (0.68–1.66)	.80
NR				
ULR				
CRP	1.9 (1.15–3.1)	.012	0.77 (0.32–1.9)	.56
NR				
ULR				
Concurrent chemotherapy	0.9 (0.55–1.53)	.74		
1				
2				
Consolidate chemotherapy	1.14 (0.77–1.68)	.52		
No				
Yes				
Myelosuppression	1.07 (0.72–1.6)	.74		
III–IV°				
I–II°				
Metastases	3.94 (2.5–6.3)	**.000**	3.45 (2.1–5.6)	**.000**
Yes				
No				
Recent efficacy	5.88 (3.3–10.5)	**.000**	2.93 (1.59–5.36)	**.001**
SD/PD				
CR/PR				
LabBM score	2.71 (1.7–4.2)	**.000**	2.75 (1.14–7)	**.03**
H				
M				
L				

Bold values indicate that the difference is significant, with a *P*-value <.05.

ALB = albumin, CRP = C-reactive protein, CR/PR = complete response/partial response, Hb = hemoglobin, H-LabBM = high LabBM score, HR = hazard ratios, KPS score = Karnofsky Performance Status, LDH = lactate dehydrogenase, L-LabBM = low LabBM score, M-LabBM = middle LabBM score, PLT = platelets.

#### 3.3.2. Progression-free survival

The median PFS of the 3 groups of L-LabBM, M-LabBM, and H-LabBM was 17, 12 months, and 7.5 months, respectively, with the specific statistical significance (*P* = .00) (Fig. 1 upper). Univariate COX regression analysis showed that age > 65 years, pretreatment metastasis, failure to achieve CR or PR after treatment, higher T stage, N+, higher TNM stage, lower serum albumin level, lower KPS score, lower Hb, higher LDH, and higher LabBM score were associated with poorer PFS (*P* < .05), while tumor length, tumor location, myelosuppression, degree of myelosuppression, gender, total radiotherapy dose, number of concurrent chemotherapy, number of consolidation chemotherapy, and chemotherapy regimen were not significantly correlated with patient PFS (*P* > .05). Multifactorial COX regression analysis showed that age > 65 years (HR 1.6 [1.06–2.5] *P* = .024), lower KPS score (HR 1.74 [1.2–2.5] *P* = .003), pretreatment metastasis (HR 5.85 [3.5–9.77] *P* = .000), and failure to achieve CR or PR after treatment (HR 3.7 [1.98–7] *P* = .000) were independent high risk factors for PFS in patients (Table 5).

**Table 5:** Univariate and multivariate analysis of profession free survival based on clinicopathological characteristics (N = 150).

Characteristics	Univariable		Multivariable	
	HR with 95% CI	*P*-value	HR with 95% CI	*P*-value
Age (yr)	1.5(1.03–2.2)	**.035**	1.6 (1.06–2.5)	**.024**
>65				
≤65				
Gender	0.66 (0.4–1.2)	.14		
Male				
Female				
Location	1.0 (0.79 1.27)	.97		
Upper				
Middle				
Down				
KPS	1.48 (1.07–2.04)	**.019**	1.74 (1.2–2.5)	**.003**
<80				
≥80				
Chemotherapy regimen	1.2 (0.81 1.74)	.39		
TP				
RP				
Lymph node metastasis (N-stage)	1.68 (1.15–2.6)	**.008**	1.25 (0.81–1.92)	.32
N0				
N1				
N2				
N3				
Tumor invasion (T stage)	1.77 (1.25–2.5)	**.001**	1.1 (0.59–1.89)	.84
T2				
T3				
T4a				
TNM stage	1.6 (1.18–2.2)	**.003**	1.1 (0.64–1.8)	.78
II				
III				
IVa				
Length	0.82 (0.52–1.3)	.39		
<5 cm				
≥5 cm				
Dose	1.36 (0.56–3.4)	.5		
<60 Gy				
≥60 Gy				
ALB	1.96 (1.1–3.5)	**.02**	0.95 (0.51–1.75)	.86
NR				
ULR				
LDH	1.8 (1.07–2.9)	**.025**	1.18 (0.66–2.1)	.58
NR				
ULR				
PLT	0.43 (0.11–176)	.24		
NR				
ULR				
Hb	1.5 (1.04–2.23)	**.03**	0.9 (0.6–1.4)	.71
NR				
ULN				
CRP	1.4 (0.85–2.35)	.18		
NR				
ULN				
Concurrent chemotherapy	1.08 (0.64–1.8)	.78		
1				
2				
Consolidate chemotherapy	1.11 (0.76–1.6)	.58		
No				
Yes				
Bone marrow suppression	1.26 (0.85–1.88)	.26		
III–IV°				
I–II°				
Metastases	6.03 (3.74–9.7)	**.000**	5.85 (3.5–9.8)	**.000**
Yes				
No				
Recent efficacy	6.42 (3.6–11.5)	**.000**	3.7 (1.98–7.0)	**.000**
SD/PD				
CR/PR				
LabBM score	2 (1.3–3.1)	**.002**	1.67 (0.97–2.9)	.06
H				
M				
L				

Bold values indicate that the difference is significant, with a *P*-value <.05.

ALB = albumin, CRP = C-reactive protein, CR/PR = complete response/partial response, Hb = hemoglobin, H-LabBM = high LabBM score, HR = hazard ratios, KPS score = Karnofsky Performance Status, LDH = lactate dehydrogenase, L-LabBM = low LabBM score, M-LabBM = middle LabBM score, PLT = platelets.

### 3.4. The training group nomogram based on the LabBM Score Risk Model

**Figure 2.**
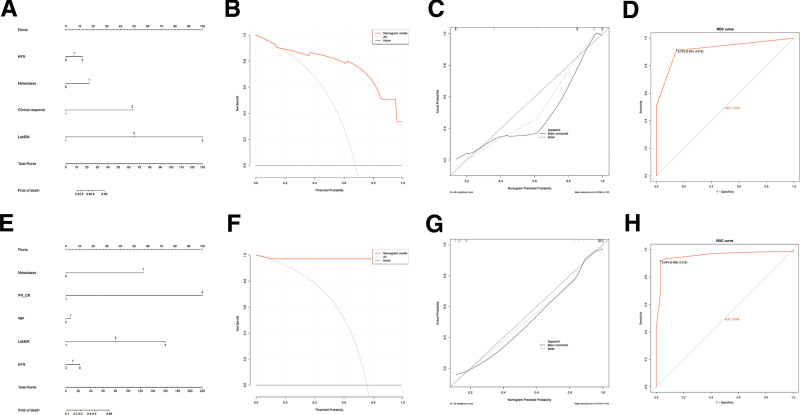
Clinical utility analysis of the nomogram constructed based on LabBM and the ROC curve of prognostic evaluation value for training cohorts OS and PFS (n = 150). (A–D) Nomogram, DCA, calibration curve, and ROC for training cohorts OS. (E–G) Nomogram, DCA, calibration curve, and ROC for training cohorts PFS. OS = overall survival, PFS = progression-free survival, ROC = receiver operating characteristic.

Risk models were constructed based on independent factors affecting OS and PFS identified in the multivariate analysis. The independent prognostic factors for OS included KPS status, LabBM score, recent treatment response, and pretreatment metastasis, while those for PFS included age, KPS status, pretreatment metastasis, recent treatment response, and LabBM score. Additionally, the clinical utility of the risk models was further evaluated using decision curve analysis curves and ROC curves, which confirmed that higher LabBM scores were associated with worse OS and PFS, consistent with the findings of the multivariate Cox regression analysis. Next, we used a nomogram to predict individual patient OS and PFS rates. The independent prognostic factors identified in the multivariate analysis, including LabBM score, pretreatment metastasis, failure to achieve CR/PR posttreatment, and KPS score, were incorporated into the nomogram (Fig. 2A–C, E–G). The calibration curve for the nomogram indicated a C-index of 0.956 for OS and 0.92 for PFS, demonstrating a high concordance between the predicted and actual survival probabilities.

### 3.5. Efficacy test of prognosis evaluation in the training group

**Figure 3.**
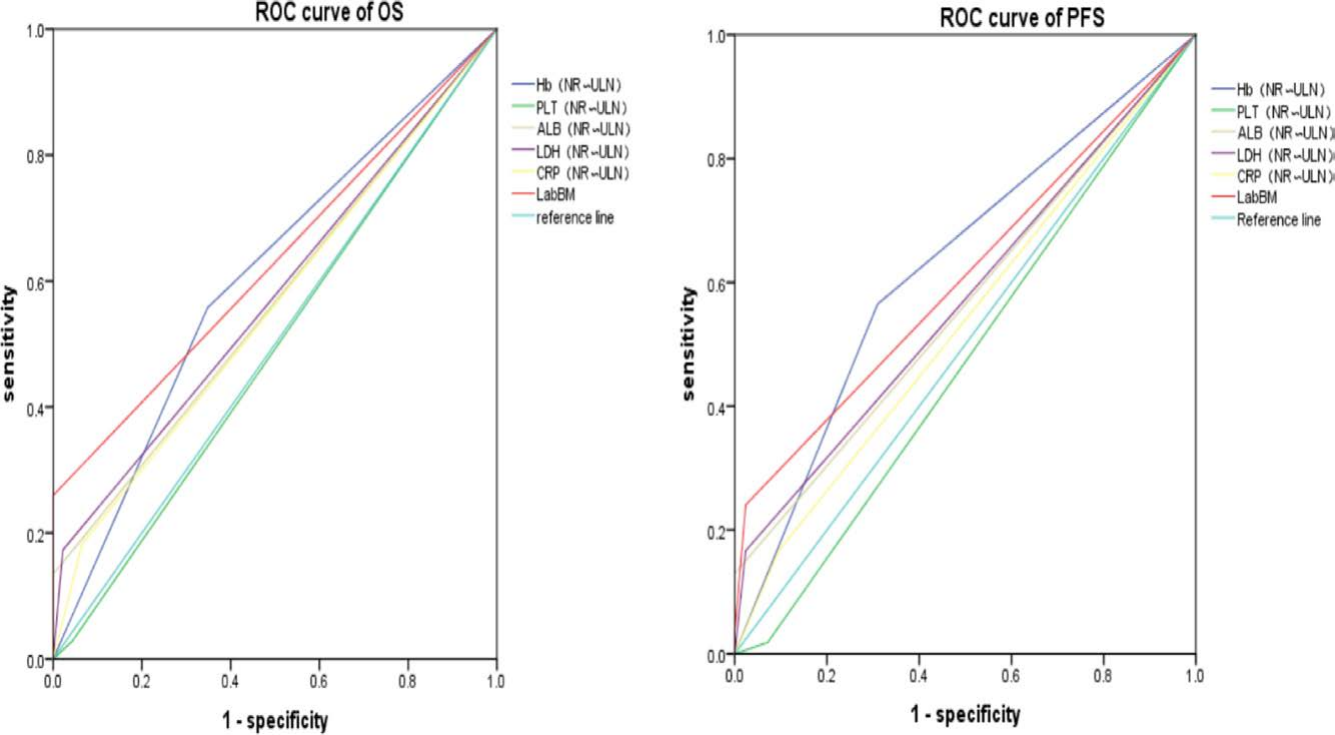
The AUC area of the each parameter in the prognostic assessment of training cohorts OS and PFS (n = 150). The AUC of LabBM scores predicting OS and PFS were 0.63 (95% CI: 0.54–0.72, *P* = .011) and 0.61 (95% CI: 0.52–0.70, *P* = .039). The AUC of OS and PFS predicted by PLT was 0.49 (95% CI: 0.39–0.59, *P* = .89) and 0.47 (95% CI: 0.37–0.58, *P* = .62). The AUC of OS and PFS predicted by ALB was 0.57 (95% CI: 0.47–0.76, *P* = .19) and 0.56 (95% CI: 0.47–0.66, *P* = .22). The AUC of LDH was 0.58 (95% CI:0.48–0.67, *P* = .14) and 0.57 (95% CI: 0.48–0.67, *P* = .18). The AUC for CRP was 0.56 (95% CI: 0.46–0.66, *P* = .25) and 0.54 (95% CI: 0.44–0.64, *P* = .5), and the AUC for OS and PFS predicted by Hb was 0.61 (95% CI: 0.51–0.70. *P* = .04) and 0.63 (95% CI: 0.53–0.73, *P* = .015). ALB = albumin, AUC = area under the ROC curve, Hb = hemoglobin, LDH = lactate dehydrogenase, OS = overall survival, PFS = progression-free survival.

Based on the OS and PFS data of the enrolled patients, ROC curve analyses were conducted using death as the endpoint variable at the end of the follow-up period. The analyses assessed the prognostic efficacy of the LabBM score risk model, LabBM score, ALB, Hb, PLT, LDH, and CRP in predicting patient outcomes. In the ROC analysis, the AUC values for OS and PFS predicted by the LabBM score risk model were 0.92 (95% CI: 0.86–0.97, *P* = .00) and 0.956 (95% CI: 0.88–0.98, *P* = .000), respectively. The AUC values for OS and PFS predicted by the LabBM score alone were 0.63 (95% CI: 0.54–0.72, *P* = .011) and 0.61 (95% CI: 0.52–0.70, *P* = .039), respectively. The AUC values for OS and PFS predicted by PLT were 0.49 (95% CI: 0.39–0.59, *P* = .89) and 0.47 (95% CI: 0.37–0.58, *P* = .62), respectively. The AUC values for OS and PFS predicted by ALB were 0.57 (95% CI: 0.47–0.76, *P* = .19) and 0.56 (95% CI: 0.47–0.66, *P* = .22), respectively. The AUC values for OS and PFS predicted by LDH were 0.58 (95% CI: 0.48–0.67, *P* = .14) and 0.57 (95% CI: 0.48–0.67, *P* = .18), respectively. The AUC values for OS and PFS predicted by CRP were 0.56 (95% CI: 0.46–0.66, *P* = .25) and 0.54 (95% CI: 0.44–0.64, *P* = .5), respectively. The AUC values for OS and PFS predicted by Hb were 0.61 (95% CI: 0.51–0.70, *P* = .04)and 0.63 (95% CI: 0.53–0.73, *P* = .015), respectively. In summary, the AUC of the LabBM risk model was significantly higher than that of the other individual parameters in predicting both OS and PFS, highlighting its superior prognostic value (Fig. 2(D, H) and Fig. 3).

## 4. Validation group results

### 4.1. The validation group characteristics

In accordance with the established inclusion and exclusion criteria, a total of 154 patients were recruited into the validation cohort, which included 113 males and 41 females, with a median age of 71 years (range: 37–80 years) at the time of diagnosis. Throughout the follow-up period, 10 patients were excluded due to accidental deaths: specifically, 2 from cerebral hemorrhage, 1 from myocardial infarction, 2 from esophageal cancer bleeding, 2 from esophagotracheal fistula, 1 from liver cirrhosis, and 2 from other acute conditions. This left a final cohort of 144 patients who completed the follow-up. The median length of the primary esophageal lesion in these patients was 6 cm (range: 2.5–17 cm). According to the AJCC 8th edition clinical TNM staging system,^[21]^ the distribution of patients by stage was as follows: stage I, 2.7% (4/144); stage II, 26.3% (38/144); stage III, 31.25% (45/144); and stage IVA, 39.5% (57/144).

Among the enrolled patients, 35 completed 2 cycles of induction chemotherapy prior to CCRT, utilizing the same chemotherapy regimen as that administered during the concurrent radiotherapy phase. Additionally, 20 patients were unable to tolerate the toxicities associated with chemotherapy during CCRT and either received only 1 cycle or did not undergo concurrent chemotherapy at all. Following the completion of the CCRT treatment protocol, 77 patients received 2 to 4 cycles of consolidation chemotherapy, adhering to the same regimen used during CCRT. Regarding performance status, 8 patients had a KPS score below 80, while 136 had a KPS score above 80. Further details are provided in Table 6.

**Table 6:** Validation group baseline characteristics, treatment, and laboratory data according to LabBM score (N = 144).

Characteristics	L-LabBM (103)	M-LabBM (30)	H-LabBM (11)	*P*
Gender				**.06** **†**
Male	72	27	9	
Female	31	3	2	
Age (yr)				.28†
≥65	79	21	7	
<65	24	9	4	
KPS				.60†
<80	6	2	0	
>80	97	28	11	
T stage				.04†
T1	4	0	0	
T2	20	1	1	
T3	40	18	3	
T4a	39	11	7	
N stage				.002†
N0	53	10	3	
N1	43	12	4	
N2	6	7	4	
N3	1	1	0	
cTNM stage				.04†
I	4	0	0	
II	33	4	1	
III	27	15	3	
IVA	39	11	7	
Length				.94†
≥5 cm	75	19	9	
<5 cm	28	10	2	
PLT				.22†
NR	101	26	11	
>ULN	2	4	0	
ALB				
NR	96	20	8	**.05** **†**
>ULN	13	8	3	
Hb				
NR	86	11	7	**.016** **†**
>ULN	17	19	4	
LDH				
NR	91	16	4	**.00** **†**
>ULN	12	14	7	
CRP				
NR	96	20	5	**.000** *
>ULN	7	10	6	

Bold values indicate that the difference is significant, with a *P*-value <.05.

ALB = albumin count, Chemo = chemotherapy, CRP = C-reactive protein, cTNM stage = clinical TNM stage, LDH = lactate dehydrogenase, N+ = Lymph node positive, N0 =Lymph node negative, NR = normal rate, PLT = platelet count, RP =Raltitrexed + cisplatin; TP =Docetaxel + cisplatin, ULN = upper limit of normal, ZPS = Zubrod⁃ECOG⁃WHO performance status.

* Chi-square test.

† Fisher exact test.

### 4.2. The relationship between LabBM score and clinicopathological characteristics in the validation group

Through the statistical analysis between LabBM score and clinicopathological factors, we found that the constitutive parameters of LabBM score also showed no obvious correlation between PLT and LabBM, and the remaining parameters were significantly correlated with LabBM composition (*P* was <.05). In addition, the In addition LabBM score was significantly correlated with T, N, and TNM stage (*P* < .05), but there was no significant correlation with age, gender, and KPS score (all *P* > .05) (Table 6).

### 4.3. Prognosis in the validation cohort

#### 4.3.1. OS

**Figure 4.**
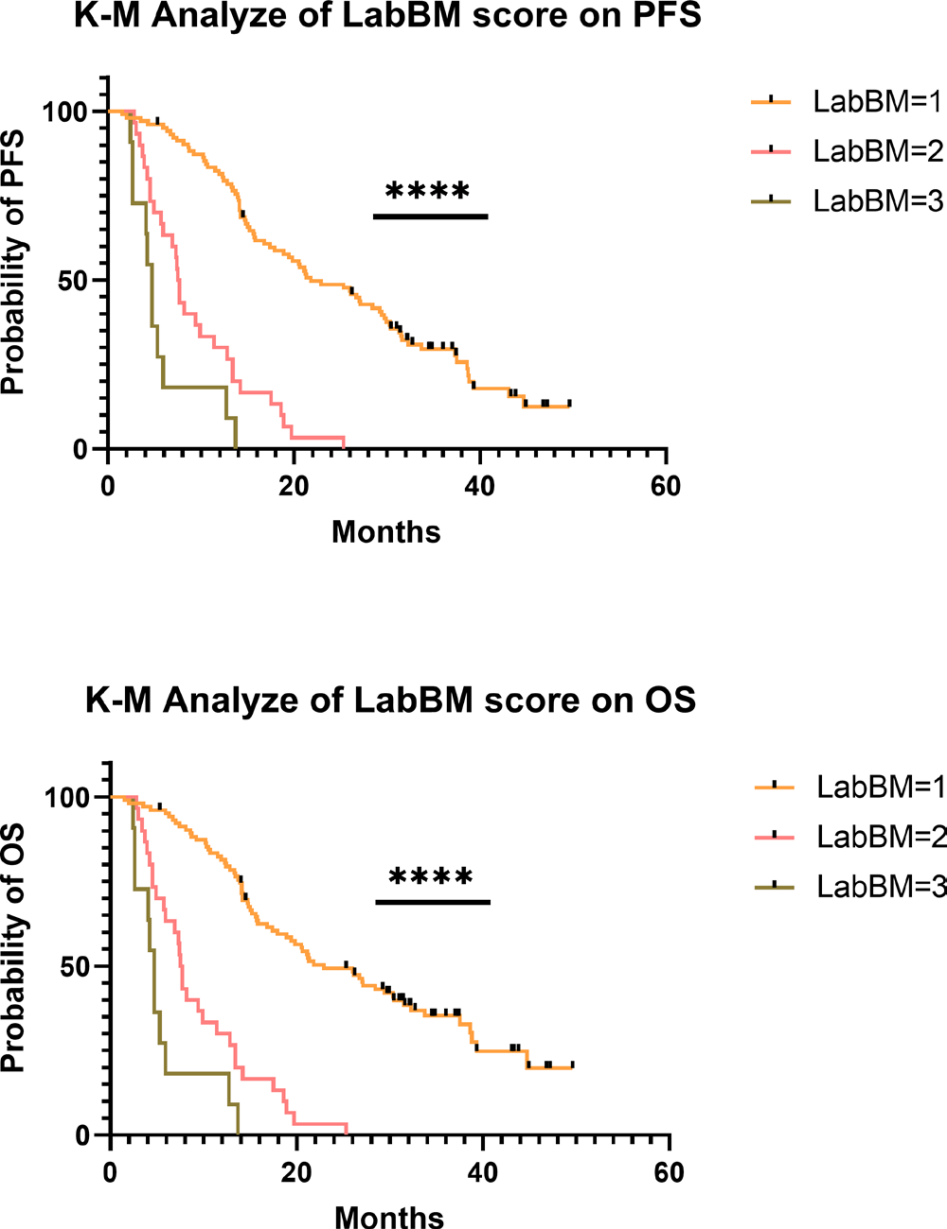
Differences in survival of different groups of LabBM score in the validation cohorts (n = 144).

In the validation cohort, patients were stratified into 3 groups based on their LabBM scores, with 103 patients in the low LabBM group (0–1), 30 patients in the medium LabBM group (1.5–2), and 11 patients in the high LabBM group (2.5–3.5). Analysis of OS showed a clear trend of decreasing median OS with increasing LabBM scores: 27.9 months for the low LabBM group, 13.1 months for the medium LabBM group, and 9.3 months for the high LabBM group. This trend was consistent with the findings from the training cohort. A significant difference in OS was observed across the 3 groups (*P* = .000) (Fig. 4, lower panel). Thus, a higher LabBM score was confirmed to be associated with worse OS (*P* < .05).

The impact of LabBM on OS was further validated using univariate and multivariate Cox regression analyses. Univariate Cox analysis indicated that failure to achieve a complete response/partial response (CR/PR), advanced T stage, later TNM stage, lower serum albumin level, higher LDH level, and higher LabBM score were all associated with worse OS (*P* < .05). In contrast, tumor length, tumor location, age, gender, total radiotherapy dose, concurrent chemotherapy, and chemotherapy regimen did not show a significant correlation with OS (*P* > .05). Multivariate Cox regression analysis revealed that a higher LabBM score (HR 3.39, 95% CI: 2.36–4.81, *P* = .000), advanced T stage (HR 0.667, 95% CI: 0.46–0.96, *P* = .03), and failure to achieve CR/PR after treatment (HR 7.16, 95% CI: 3.7–13.82, *P* = .001) were independent prognostic factors for OS (Table 7).

**Table 7:** Validation group univariate and multivariate analysis of overall survival based on clinicopathological characteristics (N = 144).

Characteristics	Univariable		Multivariable	
	HR with 95% CI	*P*-value	HR with 95% CI	*P*-value
Age (yr)	0.60 (0.35–1.05)	.07		
≤65				
>65				
Gender	1.14 (0.67–1.95)	.62		
Male				
Female				
KPS	1.81 (0.71–4.66)	.22		
<80				
≥80				
Chemotherapy regimen	0.75 (0.46–1.4)	.29		
TP				
RP				
Lymph node metastasis (N-stage)	1.06 (0.74–1.51)	.759		
N0				
N1				
N2				
N3				
Tumor invasion (T stage)	0.64 (0.43–0.94)	**.022**	0.67 (0.46–0.96)	**.03**
T1				
T2				
T3				
T4				
TNM stage	2.37 (1.18–5.0)	**.04**	2.08 (1.11–3.91)	**.023**
II–III				
IVa				
Length	0.73 (0.44–1.2)	.21		
<5 cm				
≥5 cm				
Dose	1.21 (0.64–2.28)	.56		
<60 Gy				
≥60 Gy				
ALB	0.93 (0.54–1.58)	.78		
NR				
ULR				
LDH	3.36 (1.9–5.8)	**.00**	2.68 (1.65–4.32)	**.00**
NR				
ULR				
PLT	0.55 (0.95–1.54)	.52		
NR				
ULR				
Hb	1.84 (1.08–3.56)	**.026**	1.75 (1.09–2.78)	**.018**
NR				
ULR				
CRP	1.01 (0.55–1.88)	.97		
NR				
ULR				
Concurrent chemotherapy	1.04 (0.55–1.96)	.90		
1				
2				
Consolidate chemotherapy	1.06 (0.70–1.63)	.76		
No				
Yes				
Metastases	0.54(0.137–2.08)	.34		
Yes				
No				
Recent efficacy	12.7(6.0–26.99)	**.000**	7.16 (3.7–13.82)	**.00**
SD/PD				
CR/PR				
LabBM score	3.82(2.6–5.68)	**.000**	3.4 (2.36–4.8)	**.00**
H				
M				
L				

Bold values indicate that the difference is significant, with a *P*-value <.05.

ALB = albumin, CRP = C-reactive protein, CR/PR = complete response/partial response, Hb = hemoglobin, H-LabBM = high LabBM score, HR = hazard ratios, KPS score = Karnofsky Performance Status, LDH = lactate dehydrogenase, L-LabBM = low LabBM score, M-LabBM = middle LabBM score, PLT = platelets.

#### 4.3.2. Profession free survival

In the validation cohort, the median PFS for patients in the L-LabBM, M-LabBM, and H-LabBM groups was 21.3, 7.7, and 4.7 months, respectively. A significant difference in PFS was observed among the 3 groups, and this difference was statistically significant (*F* = 34.6, *P* = .00) (Fig. 4 upper).

Univariate and multivariate Cox regression analyses were performed to further validate the risk factors affecting PFS. Univariate Cox analysis showed that pretreatment metastasis, failure to achieve CR/PR after treatment, worse N stage, lower Hb, higher LDH, and higher LabBM score were all associated with poorer PFS (*P* < .05 for all). However, there was no significant association with tumor length, tumor location, gender, total radiotherapy dose, number of concurrent chemotherapy cycles, number of consolidation chemotherapy cycles, or chemotherapy regimen (*P* > .05 for all). Multivariate Cox regression analysis of the identified risk factors for PFS demonstrated that pretreatment metastasis (HR 0.25, 95% CI: 0.08–0.73, *P* = .012), failure to achieve CR/PR after treatment (HR 14.1, 95% CI: 6.92–28.4, *P* = .000), and higher LabBM score (HR 3.82, 95% CI: 2.64–5.53, *P* = .00) were independent prognostic factors for PFS (Table 8).

**Table 8:** Validation group univariate and multivariate analysis of profession free survival based on clinicopathological characteristics (N = 144).

Characteristics	Univariable		Multivariable	
	HR with 95% CI	*P*-value	HR with 95% CI	*P*-value
Age (yr)	0.95 (0.62–1.45)	.80		
>65				
≤65				
Gender	0.79 (0.51–1.21)	.28		
Male				
Female				
KPS	0.83 (0.38–1.78)	.63		
<80				
≥80				
Chemotherapy regimen	0.87 (0.52–1.32)	.52		
TP				
RP				
Lymph node metastasis (N-stage)	1.35 (1.05–1.73)	**.02**	1.09 (0.85–1.43)	.47
N0				
N1				
N2				
N3				
Tumor invasion (T stage)	1.03 (0.83–1.29)	.78		
T1				
T2				
T3				
T4a				
TNM stage	1.42 (0.94–3.13)	.09		
II				
III				
IVa				
Length	1.04 (0.69–1.57)	.85		
<5 cm				
≥5 cm				
Dose	1.04 (0.66–1.64)	.87		
<60 Gy				
≥60 Gy				
ALB	1.56 (0.98–2.48)	**.06**		
NR				
ULR				
LDH	3.07 (2.02–4.67)	**.00**	2.1 (1.3–3.4)	**.002**
NR				
ULR				
PLT	1.58 (0.69–3.6)	.28		
NR				
ULR				
Hb	1.68 (1.09–2.57)	**.017**	1.74 (1.09–2.75)	**.019**
NR				
ULN				
CRP	1.34 (0.82–3.19)	.25		
NR				
ULN				
Concurrent chemotherapy	0.95 (0.56–1.59)	.84		
1				
2				
Consolidate cheotherapy	1.17 (0.81–1.68)	.34		
No				
Yes				
Metastases	2.52 (1.02–6.2)	**.046**	0.25 (0.08–0.74)	**.012**
Yes				
No				
Recent efficacy	9.76 (5.4–17.9)	**.000**	14.1 (6.92–28.4)	**.00**
SD/PD				
CR/PR				
LabBM score	3.88 (2.85–5.2)	**.00**	3.82 (2.64–5.5)	**.00**
H				
M				
L				

Bold values indicate that the difference is significant, with a *P*-value <.05.

ALB = albumin, CRP = C-reactive protein, CR/PR = complete response/partial response, Hb = hemoglobin, H-LabBM = high LabBM score, HR = hazard ratios, KPS score = Karnofsky Performance Status, LDH = lactate dehydrogenase, L-LabBM = low LabBM score, M-LabBM = middle LabBM score, PLT = platelets.

### 4.4. The validation group constructed the nomogram and the prognostic efficacy test of the risk model based on the LabBM score

**Figure 5.**
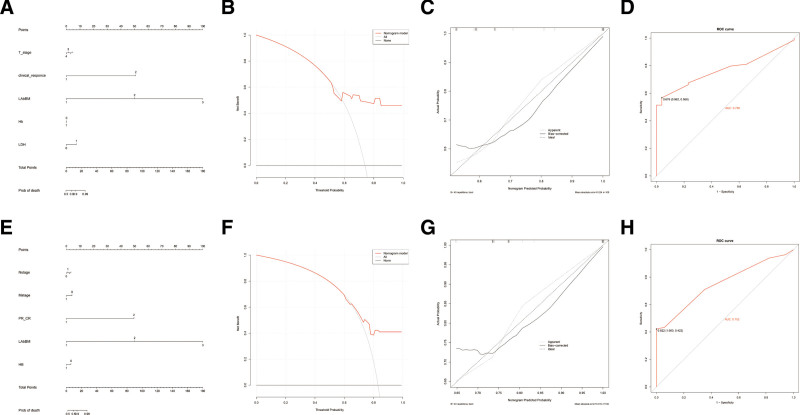
Clinical utility analysis of the nomogram constructed based on LabBM and the ROC curve of prognostic evaluation value for validation cohorts OS and PFS (n = 144). (A–D) Nomogram, DCA, calibration curve, and ROC for validation cohorts OS. (E–G) Nomogram, DCA, calibration curve, and ROC for validation cohorts PFS. OS = overall survival, PFS = progression-free survival, ROC = receiver operating characteristic.

We reconstructed a nomogram based on the LabBM score to predict individual patient OS and PFS. The nomogram incorporated independent prognostic factors identified through multivariate analysis, including LabBM score, recent treatment response, pretreatment metastasis, and T stage. Consistent with the multivariate Cox regression analysis, higher LabBM scores, advanced T stage, and failure to achieve CR/PR after treatment were associated with poorer OS and PFS outcomes. The calibration curves of the nomogram demonstrated a strong correlation between predicted and actual survival rates, with C-index values of 0.768 for OS and 0.752 for PFS, aligning well with the results from the training cohort (Fig. 5A–C and E–G).

Further evaluation of the nomogram’s prognostic performance at the end of the follow-up period involved ROC curve analysis, using death as the endpoint variable. This analysis assessed the prognostic efficacy of the LabBM score risk model, LabBM score, ALB, Hb, PLT, LDH, and CRP in the validation cohort. The AUC values for predicting OS and PFS were as follows:

- LabBM score risk model: 0.77 (95% CI: 0.71–0.89, *P* = .000) for OS and 0.75 (95% CI: 0.68–0.90, *P* = .000) for PFS.
- LabBM score alone: 0.66 (95% CI: 0.57–0.76, *P* = .005) for OS and 0.67 (95% CI: 0.57–0.77, *P* = .007) for PFS.
- PLT: 0.53 (95% CI: 0.42–0.64, *P* = .64) for OS and 0.525 (95% CI: 0.40–0.65, *P* = .69) for PFS.
- ALB: 0.53 (95% CI: 0.42–0.64, *P* = .608) for OS and 0.55 (95% CI: 0.44–0.67, *P* = .4) for PFS.
- LDH: 0.59 (95% CI: 0.48–0.69, *P* = .12) for OS and 0.61 (95% CI: 0.51–0.72, *P* = .07) for PFS.
- CRP: 0.52 (95% CI: 0.41–0.64, *P* = .66) for OS and 0.56 (95% CI: 0.38–0.63, *P* = .99) for PFS.
- Hb: 0.59 (95% CI: 0.48–0.69, *P* = .14) for OS and 0.50 (95% CI: 0.38–0.63, *P* = .998) for PFS.

**Figure 6.**
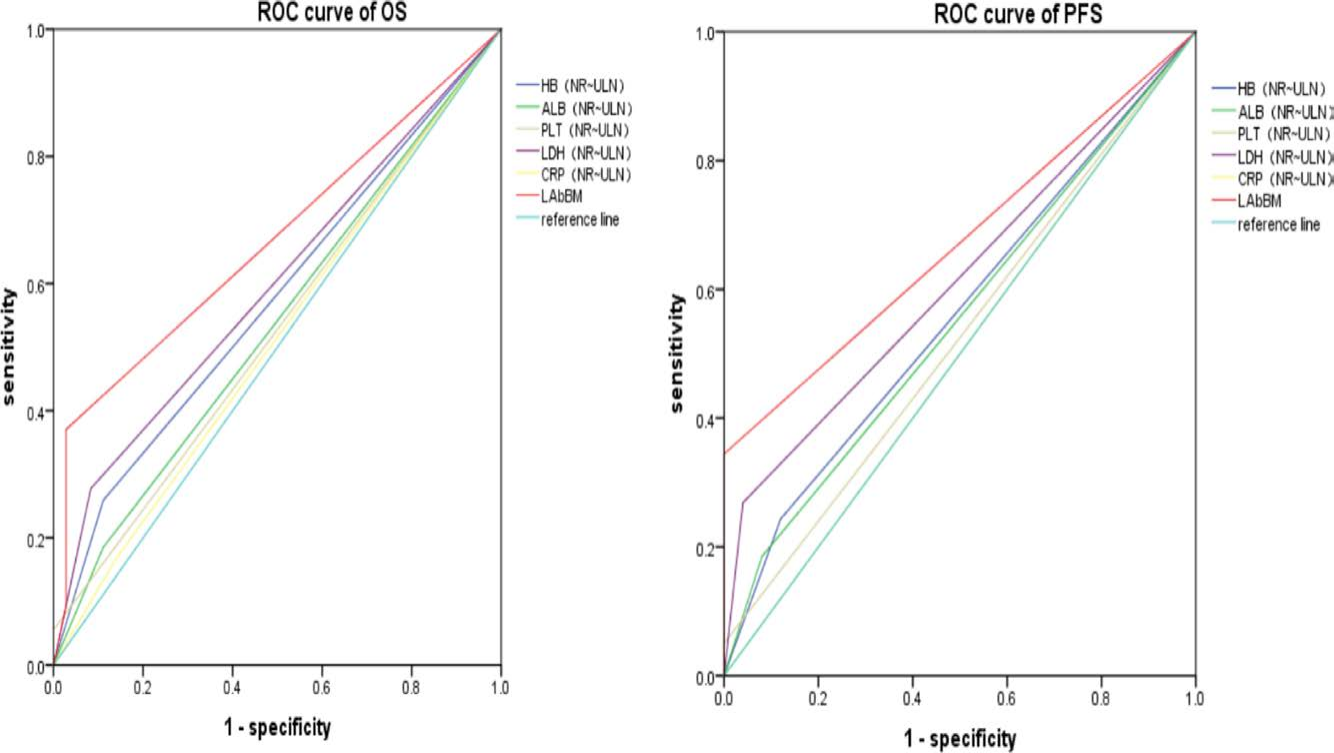
The AUC area of the each parameter in the prognostic assessment ofvalidation cohorts OS and PFS (n = 144). The AUC for the LabBM score in predicting OS and PFS was 0.66 (95% CI: 0.57–0.76, *P* = .005) and 0.67 (95% CI: 0.57–0.77, *P* = .007), respectively. The AUC for PLT in predicting OS and PFS was 0.53 (95% CI: 0.42–0.64, *P* = .64) and 0.525 (95% CI: 0.40–0.65, *P* = .69), respectively. The AUC for ALB in predicting OS and PFS was 0.53 (95% CI: 0.42–0.64, *P* = .608) and 0.55 (95% CI: 0.44–0.67, *P* = .4), respectively. The AUC for LDH was 0.59 (95% CI: 0.48–0.69, *P* = .12) and 0.61 (95% CI: 0.51–0.72, *P* = .07), respectively. The AUC for CRP was 0.52 (95% CI: 0.41–0.64, *P* = .66) and 0.56 (95% CI: 0.38–0.63, *P* = .99), respectively. The AUC for Hb in predicting OS and PFS was 0.59 (95% CI: 0.48–0.69, *P* = .14) and 0.5 (95% CI: 0.38–0.63, *P* = .998), respectively. ALB = albumin, AUC = area under the ROC curve, CRP = C-reactive protein, Hb = hemoglobin, LDH = lactate dehydrogenase, OS = overall survival, PFS = progression-free survival, PLT = platelets, ROC = receiver operating characteristic.

These results are illustrated in Figures 5D,H and 6. In summary, the LabBM risk model demonstrated significantly higher AUC values compared to the other parameters in predicting both OS and PFS, thereby highlighting its superior prognostic value.

## 5. Discussion

With advancements in tumor treatment, traditional TNM staging and histological classification no longer suffice for predicting tumor efficacy in clinical practice.^[22]^ Numerous laboratory blood parameters, including mean platelet volume, HB, CRP to albumin ratio, neutrophil-to-lymphocyte ratio, platelet-to-lymphocyte ratio, monocyte-to-lymphocyte ratio, systemic immune-inflammation index, and prognostic nutritional index, have been shown to be associated with tumor progression and unfavorable prognosis in ESCC.^[23–26]^ The Osaka prognostic score and BAN score, have also shown prognostic value in ESCC. The Osaka prognostic score, based on ALB, CRP, and total lymphocyte count, predicted OS with a C-index of 0.68.^[21]^ The BAN score, incorporating BMI, ALB, and neutrophil-to-lymphocyte ratio, was an independent prognostic factor for DFS and OS.^[27]^ These findings highlight the importance of integrating multiple parameters for more accurate prognostic assessment in ESCC patients undergoing CCRT. Two recent studies, including 1 by Lichun Wu et al, constructed a prognostic model for ESCC using Random Survival Forest based on patients’ hematological indicators and clinical pathological parameters. The C-index of this model was 0.746, with 3-year and 5-year AUC values of 0.761 and 0.771, respectively, demonstrating better prognostic discrimination than the traditional TNM staging system.^[22]^ Another study by Fuzhong Xue et al developed and validated ESCC risk prediction models based on an endoscopic screening program. Model A had Harrell C statistics of 0.80 and 0.90 in the training and validation sets, respectively, while Model B had Harrell C statistics of 0.83 and 0.91, respectively, showing good discrimination and predictive ability. These studies, employing machine learning techniques and endoscopic screening programs, can help identify high-risk patients so that they can receive more aggressive treatment.^[28]^

To enhance prognostic accuracy, researchers have incorporated additional clinical indicators, such as Hb, ALB, LDH, and CRP.^[21,29,30]^ The LabBM score, which combines these indicators along with PLT, has shown prognostic value in various tumors. Initially proposed by Berghoff et al for brain metastasis patients, it demonstrated that higher LabBM scores correlate with worse prognosis.^[18]^ Subsequent studies, including 1 by Niederer et al, validated its applicability across multiple tumor types undergoing radiotherapy.^[20]^ However, no studies have explored the LabBM score in ESCC. Our retrospective analysis of 150 inoperable ESCC patients undergoing CCRT revealed that a higher LabBM score (HR 2.75, 95% CI: 1.08–7.03, *P* = .034) independently predicted poorer OS. This finding was consistent in a validation cohort of 155 patients, with a similar hazard ratio (HR: 3.39, 95% CI: 2.36–4.81, *P* = .000). ROC curve analysis confirmed that the LabBM score (AUC: 0.661, 95% CI: 0.566–0.756, *P* = .005) outperformed individual parameters.

Despite the significant correlation between most LabBM components and prognosis, PLT showed no significant association, echoing findings by Niederer et al.^[20]^ They modified the LabBM score by removing PLT to create a new M-LabBM score, but it did not improve prognostic differentiation. Thus, PLT was retained in our study, with below-normal levels assigned 0.5 points. Further research is needed to refine PLT scoring criteria.

However, given that the AUC of the LabBM score alone was <0.75, the clinical predictive value remains debatable. Therefore, we developed a LabBM-based risk model by incorporating independent factors affecting OS and PFS, and subsequent studies on its predictive value demonstrated that the AUC for the risk model in the training cohort was 0.92 (95% CI: 0.86–0.97, *P* = .00) for OS and 0.956 (95% CI: 0.88–0.98, *P* = .000) for PFS, with C-indices of 0.956 and 0.92, respectively. This predictive value far exceeded that of the LabBM score alone. Similar conclusions were drawn in the validation cohort.

This study is the first to demonstrate the prognostic assessment value of the LabBM score in patients with inoperable esophageal cancer. This paper now reveals several significant findings: LabBM score is a prognostic marker for ESCC patients undergoing CCRT, and LabBM score, but not any other indicator, is a valuable independent prognostic factor. It has also been shown that the LabBM score can predict the prognosis of oncology patients independently of radiographic imaging, observer variability, and the health care system. Based on the hematologic indicators that make up the LabBM score, it reflects total tumor load, bone marrow reserve, inflammatory process, and cachexia. Moreover, the LabBM score has the characteristics of low cost, fast detection, and noninvasive. Based on the above analysis results, the LabBM score can be considered one of the most straightforward and most appropriate prognostic prediction tools for patients with inoperable ESCC. LabBM scores, but not any other single indicator, are useful as independent prognostic factors. The risk model based on LabBM has better prognosis prediction value. However, there are some limitations in this study: first, the data were obtained from a single-center retrospective study, which may be biased; second, this study is a retrospective analysis, and the sample size is limited by the number of cases. We did not perform a priori sample size calculation, which may limit the results; third, the number of patients enrolled in this study was small, and the role of LabBM score in esophageal cancer and even digestive system tumors has rarely been reported in the past, so the results of this study still need further confirmation in a large sample and prospective study; last, this study found that LabBM score constitutes PLT according to thirdly, the present study found that LabBM score includes PLT according to the scoring criteria, and the role of PLT in the prognosis and long-term survival of esophageal cancer is very little, so the author will refer to previous studies in future work and will adjust the scoring criteria of PLT for further research.

## 6. Conclusion

Based on the results of this study, TNM staging did not show satisfactory outcomes in both the training and validation cohorts through both univariate and multivariate Cox analysis. So the risk model based on LabBM score is a novel and effective prognosis evaluation index in inoperable ESCC patients. In the future, we will continue to focus on developing prognostic prediction models for patients with inoperable esophageal cancer. We plan to incorporate a broader range of prognostic factors, including radiomics and molecular biomarkers, to establish more comprehensive predictive models.

## Author contributions

**Conceptualization:** Lei Zhao, Liang Liang.

**Data curation:** Lei Zhao, Dan Mei, Xiaolin Ge, Yujing Shi.

**Formal analysis:** Lei Zhao, Dan Mei, Xiaolin Ge, Yujing Shi.

**Methodology:** Lei Zhao, Dan Mei, Yujing Shi, Xinchen Sun.

**Project administration:** Xinchen Sun.

**Resources:** Lei Zhao, Xiaolin Ge, Xinchen Sun.

**Software:** Lei Zhao, Dan Mei.

**Supervision:** Liang Liang, Xinchen Sun.

**Validation:** Liang Liang, Xinchen Sun.

**Visualization:** Liang Liang.

**Writing – original draft:** Lei Zhao.

**Writing – review & editing:** Liang Liang, Yujing Shi, Xinchen Sun.
